# MicroRNA-200c Affects Milk Fat Synthesis by Targeting *PANK3* in Ovine Mammary Epithelial Cells

**DOI:** 10.3390/ijms232415601

**Published:** 2022-12-09

**Authors:** Zhiyun Hao, Jiqing Wang, Yuzhu Luo, Jiang Hu, Xiu Liu, Shaobin Li, Mingna Li, Bingang Shi, Liyan Hu, Yuan Liu, Huimin Zhen

**Affiliations:** Gansu Key Laboratory of Herbivorous Animal Biotechnology, College of Animal Science and Technology, Gansu Agricultural University, Lanzhou 730070, China

**Keywords:** microRNA-200c, MECs, sheep, mammary gland, lactation

## Abstract

Milk fat is the foremost nutrient of milk and a vital indicator in evaluating milk quality. Accumulating evidence suggests that microRNAs (miRNAs) are involved in the synthesis of milk fat. The miR-200c is closely related to lipid metabolism, but little is known about its effect on the synthesis of milk fat in MECs of ewes. Herein, the effect of miR-200c on the proliferation of ovine mammary epithelial cells (MECs) and its target relationship with a predicted target gene were investigated. The regulatory effects of miR-200c on the expression of the target genes and the content of triglycerides in ovine MECs were further analyzed. The results revealed that the expression level of miR-200c was differentially expressed in both eight tissues selected during lactation and in mammary gland tissues at different physiological periods. Overexpression of miR-200c inhibited the viability and proliferation of ovine MECs, while inhibition of miR-200c increased cell viability and promoted the proliferation of ovine MECs. Target gene prediction results indicated that miR-200c would bind the 3′UTR region of pantothenate kinase 3 (*PANK3*). Overexpression of miR-200c reduced the luciferase activity of *PANK3*, while inhibition of miR-200c increased its luciferase activity. These findings illustrated that miR-200c could directly interact with the target site of the *PANK3*. It was further found that overexpression of miR-200c reduced the expression levels of *PANK3* and, thus, accelerated the synthesis of triglycerides. In contrary, the inhibitor of miR-200c promoted the expression of *PANK3* that, thus, inhibited the synthesis of triglycerides in ovine MECs. Together, these findings revealed that miR-200c promotes the triglycerides synthesis in ovine MECs via increasing the lipid synthesis related genes expression by targeting *PANK3*.

## 1. Introduction

Milk is the principal source of nutrition to the neonate, and also provides multiple nutrients in growth and development of newborns, including lactoferrin, carbohydrates, protein, fat, vitamins, minerals and fatty acids. The lactation performance of ewes affects the healthy development of lambs and, therefore, plays a key role in the economic benefits to farmers. Studies have found that the yield and ingredients of ewes’ milk mainly determine the survival rate of multiple lambs [1,2], and also significantly influence the growth rate and development of lambs during lactation [3]. Researchers found that among the factors that caused the death of multiple lambs in Romney ewes, insufficient milk yield in the early postpartum period is the main factor, which accounts for 41.7% of the deaths of multiple lambs [1]. Especially in the first 3–4 weeks of the life, milk is the major source of food [2,4]. In China, some ewes have palmary reproductive rate, including small-tailed Han sheep, Hu sheep and other domestic breeds [5,6]. Although they have an excellent lactation performance, it is still largely lower than dairy sheep such as East Friensian, Assaf and Lacaune ewes [7]. Among the heritable factors, the milk yield and milk ingredients of ewes are directly controlled by the degree of mammary gland development [8]. For the dairy industry, the ovine lactation performance also plays an important role in the sustainable development of the milk industry, especially in European and Central Asian countries, in which milk is mainly used to produce milk powder, yogurt and cheese. If the molecular mechanism regulating mammary gland development can be uncovered, it can provide theoretical guidance for the genetic improvement of lactation traits.

The microRNAs (miRNAs) are a type of non-coding RNA with a length of 18–25 nucleotides (nt) generated by endogenous genes [9]. Studies have found that miRNAs are extensively involved in the regulation of morphogenesis and cell activity of various organs of animals and plants, including proliferation, differentiation, apoptosis and death [9,10,11]. The function of miRNAs is to inhibit or degrade the expression level of the target genes at the post-transcriptional regulation process by binding to the 3′ untranslated regions (UTR) of the target genes [12]. Furthermore, miRNAs have also been reported to regulate cells activity by other ways. For example, miRNAs can inhibit the circularization of mRNA by preventing the poly (A) sequences from binding to the 5′UTR sequences of the mRNA molecule through regulating translation initiation device. The incomplete pairing between miRNA and target mRNA molecules can not only inhibit the translation level of target genes, but also undermine the stability of mRNA [12]. Moreover, studies have demonstrated that miRNA may bind to the promoter region of target genes to enhance mRNA expression [13]. In the mammalian genome, miRNAs would regulate the expression level of protein-coding genes at least 30% [10], and then participate in various stages of vital movement [9].

Studies have reported that miRNAs can affect the development and lactation of mammary gland by regulating the activity and numbers of mammary epithelial cells (MECs), and the synthesis of milk protein and milk fat in cows, goats and rats [14,15,16]. Many miRNAs have been described that regulate the mammary gland development and lactation, such as miR-152, miR-143, and miR-148. For example, Shen et al. (2019) found that overexpression of miR-152 promoted triglycerides synthesis in cow’s MECs, while silenced miR-152 inhibited triglycerides synthesis [17]. Ji et al. (2016) revealed that overexpression of miR-143 promoted apoptosis and inhibited proliferation of caprine MECs [18]. Overexpression of miR-26a decreased the expression of the genes related to milk fat synthesis by targeting *INSIG1*, thereby inhibiting fatty acid synthesis, and content of unsaturated fatty acid and triglycerides of caprine MECs [15]. These results suggest that miRNAs are an indispensable regulatory factor for regulating mammary gland development and lactation.

The miR-200 family includes miR-200a/b/c, miR-429 and miR-141, and the seed sequence of miR-200 family are highly conserved in the organism. Functional studies of miR-200c have mainly focused on tumor cells [19,20], and there are a few reports in the MECs. A study investigated by Galio et al. (2013) stated that miR-21, miR-205 and miR-200 can maintain the epithelial state and maintain the secretory phenotype of MECs [21]. The miR-200c influences FGFR-mediated epithelial proliferation during branching morphogenesis via a Vldlr-dependent mechanism [22]. Accordingly, in this study, we investigated the effect of miR-200c on the proliferation of ovine MECs and characterized the target genes for miR-200c. We also assessed the effects of miR-200c on the expression of the target genes and the content of triglycerides in the ovine MECs.

## 2. Results

### 2.1. MiR-200c Is Associated with Development and Lactation of Mammary Gland

**Figure 1:**
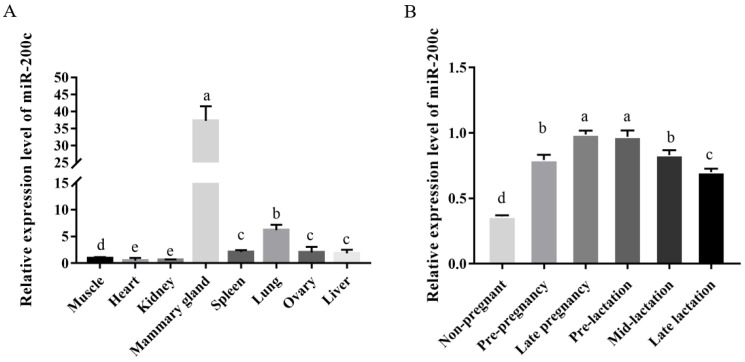
The expression level of miR-200c in the ovine eight different tissues (**A**) and the mammary gland at different developmental stages (**B**). The data are shown as mean ± SD (*n* = 3). Values with different lowercase letters are different at *p* < 0.05.

The RT-qPCR results indicated that miR-200c was widely expressed in heart, lung, liver, spleen, ovary, kidney, muscle and mammary gland, with the highest expressed in mammary gland, lung and ovary, and the lowest expression in kidney (Figure 1A). The expression level of miR-200c was the highest in mammary gland tissue, which was 63.22-fold, 20.25-fold and 6.05-fold higher than kidney, liver and lung, respectively. Moreover, the expression level of miR-200c has temporal and spatial specificity. Briefly, it had the highest expression level in the mammary gland at late pregnancy (D140), followed by the early lactation (I15), while it had the lowest expression level in the non-pregnancy period (Figure 1B). The expression level of miR-200c in mammary gland at late pregnancy was 2.73-fold higher than in the non-pregnant period (*p* < 0.05), while the expression level of miR-200c in late pregnancy was not significantly higher than that in early lactation (*p* > 0.05).

### 2.2. miR-200c Affects the Proliferation of Ovine MECs

**Figure 2:**
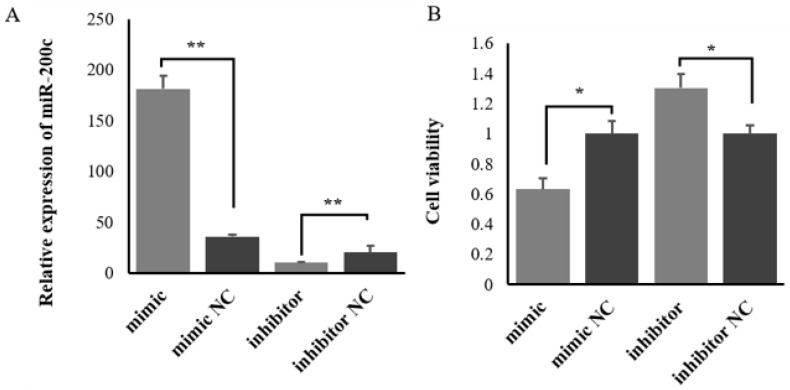
The effect of miR-200c on the viability of ovine MECs. (**A**) The relative expression levels of miR-200c when miR-200c mimic and miR-200c inhibitor were transfected into ovine MECs. (**B**) The viability of ovine MECs detected using CCK8 assay when miR-200c mimic and miR-200c inhibitor were transfected into ovine MECs. ** *p* < 0.01 and * *p* < 0.05.

The efficiency of miRNA mimic and inhibitor transfection were confirmed by RT-qPCR. The results indicated that miR-200c mimic markedly increased miR-200c expression (Figure 2A), whereas miR-200c inhibitor considerably inhibited miR-200c expression in ovine MECs (Figure 2A). The CCK8 analysis found that overexpression of miR-200c inhibited the viability of ovine MECs, while silenced miR-200c promoted cell viability (Figure 2B).

**Figure 3:**
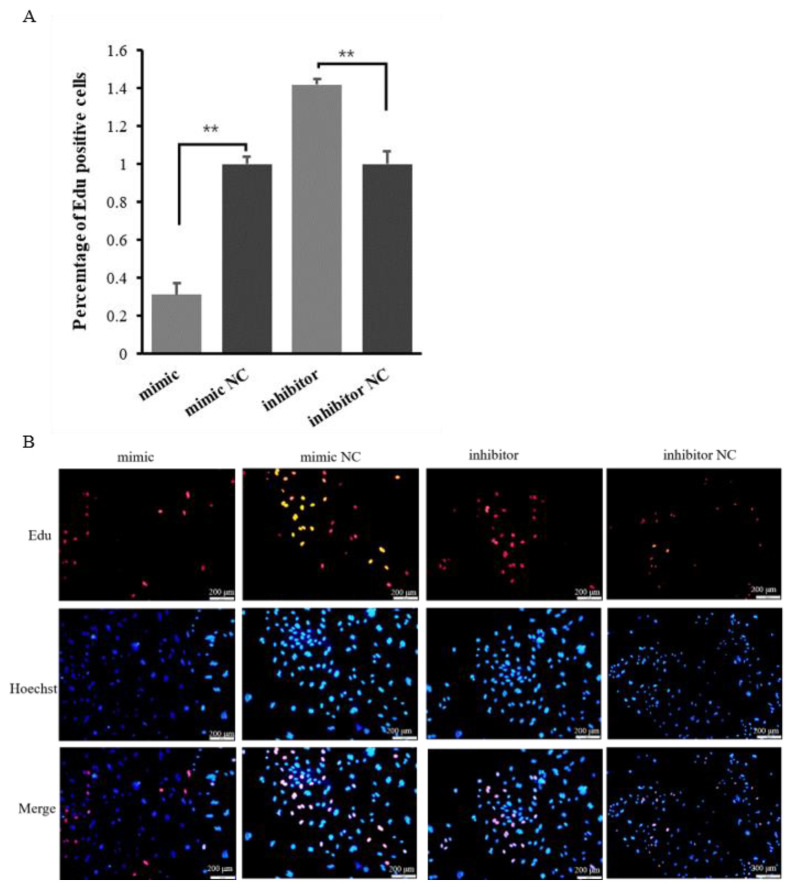
The effect of miR-200c on the proliferation of ovine MECs. (**A**) The proliferation of ovine MECs detected using Edu assay when miR-200c mimic and miR-200c inhibitor were transfected into ovine MECs. ** *p* < 0.01. (**B**) The Edu assay for detecting the proliferation of ovine MECs using a fluorescence microscope.

The Edu analysis results showed that overexpression of miR-200c reduced the number of Edu-labeled proliferated cells, while silenced miR-200c promoted the proliferation of ovine MECs (Figure 3A,B). These results indicate that miR-200c can inhibit the proliferation of ovine MECs.

### 2.3. PANK3 Is a Direct Target Gene of miR-200c

**Figure 4:**
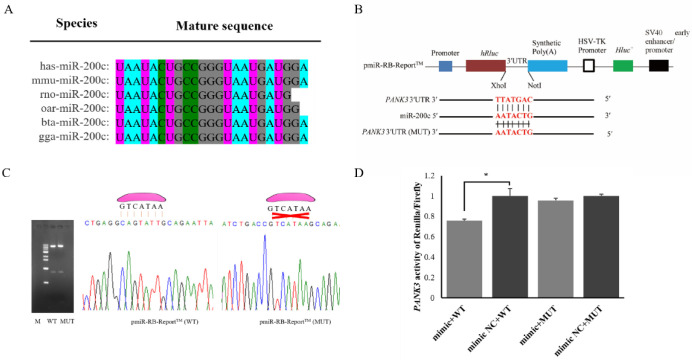
The target relationship verification of miR-200c and *PANK3* using dual luciferase reporter assay. (**A**) Homology analysis of miR-200c in different species (**B**) The structural diagram of wild-type (WT) and mutant-type (MUT) dual luciferase reporter vectors. (**C**) The figure on the left indicates the results from agarose gel electrophoresis when WT and MUT pmirR-RB-Report^TM^ vectors were digested with the restriction endonucleases NotI and Xhol. The figure on the right shows the results from Sanger sequencing. The miR-200c was indicated with a pink irregular shape above sequencing figure. (**D**) The renilla/firefly activity was measured when miR-200c mimic or miR-200c NC, and wild-type or mutated pmirR-RB-Report^TM^ vectors were co-transfected into HEK293T cells. The values represent mean ± SD (*n* = 3), * *p* < 0.05.

The sequence alignment indicated that the mature sequence of miR-200c is conserved between different species, indicating that miR-200c is highly conserved in different species (Figure 4A). The prediction results of miRanda 3.3a and TargetScan 3.1 have shown that miR-200c would target *PANK3* (Pantothenate Kinase 3) gene. Following this, dual luciferase reporter vectors were successfully constructed to verify their targeting relationship (Figure 4B). The constructed wild-type and mutant dual-luciferase vectors were subjected to digestion using restriction endonuclease XhoI/NotI, and the consequences of agarose gel electrophoresis have then shown that the foreign gene has been successfully connected to the vector (Figure 4C). Sanger sequencing results have shown that both the wild-type and mutant pmiR-RB-Report™ vectors contained expected sequences, indicating that the vectors have been successfully constructed as expected (Figure 4C). The results of dual luciferase reporter gene detection have shown that overexpression of miR-200c significantly reduced the ratio of renilla enzyme to firefly enzyme activity in the *PANK3* wild-type vector of pmiR-RB-Report™. However, there was no significant effect on the ratio in the *PANK3* mutant vector of pmiR-RB-Report™ when miR-200c was over-expressed (Figure 4D). Together, these results indicate that *PANK3* is a direct target gene of miR-200c.

### 2.4. miR-200c Affects the Milk Synthesis Process by Regulating the Expression of PANK3

**Figure 5:**
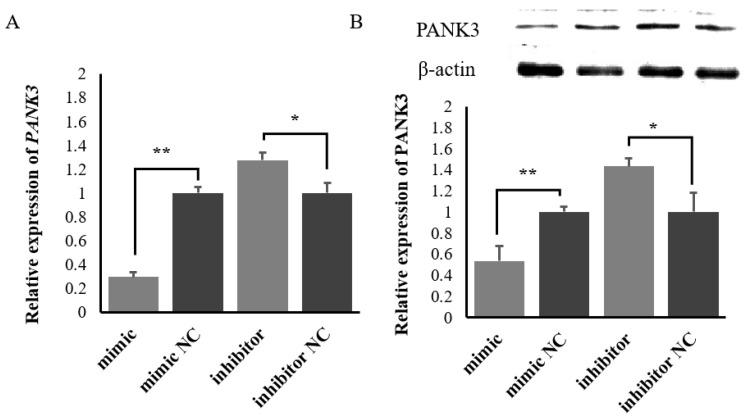
The expression of PANK3 at the mRNA and protein levels when the miR-200c mimic or the miR-200c inhibitor were transfected into ovine mammary epithelial cells (MECs). (**A**) The regulation of the mRNA level of *PANK3* by miR-200c. (**B**) The regulation of the protein level of PANK3 by miR-200c. Data are presented as mean ± SD for three replicates; ** *p* < 0.01 and * *p* < 0.05. The name of each strip from left to right was mimic, mimic NC, inhibitor and inhibitor NC.

When the miR-200c mimic, miR-200c inhibitor and corresponding NC were transfected into ovine MECs, the RT-qPCR results showed that overexpression of miR-200c significantly inhibited *PANK3* expression, while silenced miR-200c promoted *PANK3* expression (Figure 5A). The western blot results showed that overexpression of miR-200c significantly inhibited the expression of PANK3 protein, while silenced miR-200c promoted PANK3 protein expression (Figure 5B).

**Figure 6:**
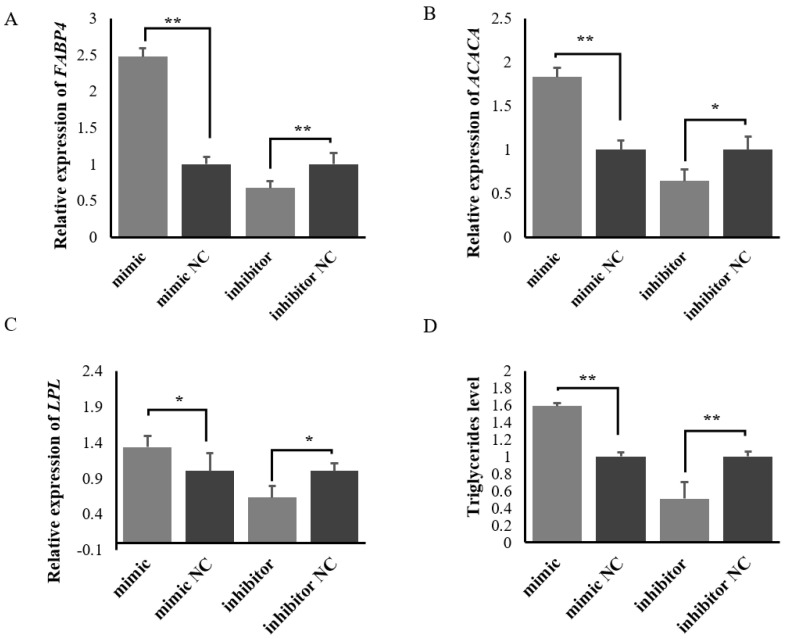
The effect of miR-200c on *FABP4* (**A**), *ACACA* (**B**) and *LPL* (**C**) and triglycerides level (**D**) in ovine MECs when the miR-200c mimic, the miR-200c inhibitor and their NC were transfected into ovine MECs. Data are presented as mean ± SD (*n* = 3). ** *p* < 0.01 and * *p* < 0.05.

A RT-qPCR results showed that overexpression of miR-200c promoted the expression levels of milk fat synthesis-related genes *FABP4*, *LPL* and *ACACA*, while silenced miR-200c inhibited the expression levels of the genes in ovine MECs (Figure 6). Triglycerides detection further showed that overexpression of miR-200c promoted triglycerides synthesis, while silenced miR-200c inhibited triglycerides synthesis in ovine MECs. These results indicate that miR-200c promotes the synthesis of triglycerides in ovine MECs.

## 3. Discussion

Since the miRNA lin-4 was initially discovered in *C. elegans* [23], an increasing number of miRNAs have been also considered to regulate mammary gland development and lactation in domestic animals [16]. As a cyclical developmental organ, the mammary gland is subjected to an extremely complex regulation process for milk synthesis, but its regulation mechanism is gradually disclosed. Up to now, studies on the effect of miRNAs on the mammary gland development and lactation have mainly been concentrated on dairy cows and dairy goats [15,16]. However, there are thousands of miRNAs found in the mammary gland for ewes, and the function of most miRNAs is still unknown.

The results indicated that miR-200c was expressed in eight tissues of our study and its expression showed significant differences in diverse tissues and mammary gland at various developmental stages. The expression level of miR-200c was the highest in mammary gland tissue. It shows that miR-200c may play important roles in ovine mammary gland development. As a popular miRNA, miR-200c has been found to be widely expressed in a variety of tissues. For example, miR-200 family has been reported to be expressed in the mammary gland of human, which then promoted a well-differentiated epithelial phenotype [24]. The miR-200c suppressed growth and induces differentiation in cancer cells, whereas in the normal mouse gland, it suppresses the expression of *BMI1* and the ability of mouse mammary repopulating units (MRUs) to develop outgrowths [25]. Notably, reduced repopulation capability has not been confirmed in the normal human breast [25]. The miR-200c protects E-cadherin from downregulation by targeting the E-box-binding zinc finger transcription factors *ZEB1* and *ZEB2*, which can suppress E-cadherin transcription [26]. Furthermore, the miR-200c enhances ovine kidney cell reprogramming into pluripotent cells by targeting *ZEB1* [27]. Guzel et al. (2021) reported that miR-200c was involved in rat liver ischaemia-reperfusion injury through oxidative stress, apoptosis and endoplasmic reticulum stress [28]. A study investigated by Wei et al. (2021) reported that the downregulation of miR-200c promotes lactate dehydrogenase A (LDHA) expression in lung, which one of the subunits of lactate dehydrogenase (LDH), participating in the process of aerobic glycolysis process by catalyzing pyruvate into lactate [29]. Taken together, these results suggest that miR-200c plays regulatory roles in many activities.

In the study, the expression of miR-200c was also different in mammary gland tissues of Small-tailed Han sheep at different developmental stages. The expression level of miR-200c has a temporal and spatial specificity. Briefly, it had the highest expression level in mammary gland at the late pregnancy, followed by the early lactation. However, it had the lowest expression level in the non-pregnancy period (Figure 1B). The higher expression of miR-200c in mammary gland tissue at late pregnancy suggests that miR-200c may be directly involved in the development of mammary glands, and may regulate milk components by affecting the process of milk synthesis. This phenomenon has also been reported in previous studies. For example, in our previous study, we also found that the expression level of miR-200c was also 4.90-fold higher in lactating mammary gland than non-lactation period [30]. In mammary glands, as another member of miR-200 family, miR-200a expression increased during mid-pregnancy through lactation, which may be stimulated by lactogenic hormone treatment of mammary epithelial cells [31]. Lactogenic hormone also induced the expression of β-casein (a marker of cell differentiation) and E-cadherin mRNA (a marker of epithelial cells) [31]. A study investigated by Li et al. (2012) [32] also found that the expression level of miR-200c in the mammary gland tissue of dairy cows during lactation was higher than that in non-lactating period. Specifically, the normalized expression of miR-200c in lactation period was 97,776 (FPKM), which was 6.66-fold of that in non-lactating period [32]. Billa et al. (2019) found that miR-200c is one of the dominant miRNAs in Holstein and Montbéliarde cows during lactation, which can regulate the synthesis of milk [33]. The miR-200 family were all expressed during pregnancy, and the expression level increased at the end of pregnancy and lactation [21]. The experimental in situ hybridization revealed that expression of the miR-200 family was mainly in luminal epithelial cells, which maintain the epithelial phenotype by inhibiting epithelial–mesenchymal transition through targeting *ZEB1* and *ZEB2*, encoding two transcriptional repressors of E-cadherin [34]. Zhang et al. (2013) also found that the expression level of the miR-200 family in the mid-lactation period was significantly higher than that in the dry period (*p* < 0.01), indicating that the miR-200 family may be involved in the regulation of the lactation process of dairy goats [35].

Many studies have demonstrated that the number and secretory activity of MECs, and the development of mammary gland directly determine milk yield and milk components in mammals [36,37]. The CCK8 and Edu detection results showed that overexpression of miR-200c in ovine MECs significantly reduced the vitality and number of ovine MECs, while silenced miR-200c promoted the viability and number of ovine MECs. These results indicate that miR-200c can inhibit the viability and number of ovine MECs. This phenomenon agreed with the previous findings that miR-200c strongly inhibits the cell proliferation of human breast cancer stem cells (BCSCs) during the initiation of tumors, which strongly suppressed the ability of normal mammary stem cells to form mammary ducts and tumor formation driven by human BCSCs in vivo [25]. Tavazoie et al. (2008) also reported that miR-200c inhibited cell proliferation and migration by targeting *ZEB* during cancer progression [34,38]. Furthermore, miR-200c expression was negatively correlated with differentiation levels in both the luminal and basal branches of the bovine mammary cell hierarchy [39]. Taken together, these suggest that miR-200c was involved in the regulation of milk yield and milk components by regulating the proliferation and viability of ovine MECs.

At the present stage, considerable research has shown that miRNA is involved in the regulation of various activities through targeting multiple genes. In this study, a total of 191 target genes were predicted for miR-200c using Targetscan 3.1 and miRanda 3.3a. Of the predicted target genes, *PANK3* attracts our attention. Firstly, in our previous studies, the expression levels of the miRNA and mRNAs were obtained in the same mammary gland tissue using small RNA-seq and RNA-seq, respectively [30,40]. The negative correlation in expression between miR-200c with *PANK3* was initially tested. Secondly, PANK3 encodes a protein belonging to the pantothenate kinase family. The pantothenate kinase inhibits the biosynthesis of coenzyme A (CoA) that is necessary for synthesis of milk fat [41]. These suggests that PANK3 may inhibit the synthesis of milk fat. Meanwhile, *PANK3* has been found to be up-regulated in mammary gland tissue during the peak-lactation period compared with the non-lactation period [40]. The gene has been also found to be down-regulated in the lactating mammary gland tissues in Small-tailed Han ewes with higher yield, and fat and protein content in milks [42]. Therefore, we finally chose *PANK3* for investigating its target relationship with miR-200c. Subsequently, the dual luciferase reporter assay was further used to identify the relationship between miR-200c and *PANK3*, and results suggests that miR-200c can target *PANK3*.

The target relationship between miR-200c with *PANK3* has been reported. The miR-200c has been found to promote papillary thyroid cancer cell proliferation, migration, and invasion of papillary thyroid cancer cell by downregulating *PTEN* [43], while overexpression of *PTEN* decreased the proliferation and differentiation of mammary epithelium in mice, and increased cell apoptosis, resulting in the death or growth postponement of newborn offspring [44].

Further studies found that overexpression of miR-200c significantly reduced the expression levels of *PANK3* and accelerated the synthesis of triglycerides. Conversely, the inhibition of miR-200c promoted the expression of *PANK3* and inhibited the synthesis of triglycerides in ovine MECs. Studies have reported that the expression of *FABP4*, *LPL* and *ACACA* were increased during the process of milk fat synthesis [45]. Similarly, another study also reported that the expression level of *LPL*, *FASN*, *ACACA*, *PLIN3* and *FABP3* related to triacylglycerol synthesis and secretion were increased in caprine MECs after overexpression *PPARγ* [46]. This result indicates that the synthesis of triglycerides is accompanied by an increase in the expression of genes related to synthesis and secretion of triacylglycerol. Our RT-qPCR results revealed that overexpression of miR-200c promoted the expression levels of milk fat synthesis-related genes *FABP4*, *LPL* and *ACACA* in ovine MECs, while silenced miR-200c inhibited the expression levels of milk fat synthesis-related genes in ovine MECs. Meanwhile, miR-200c also regulated the expression of *PANK3* in the study. The *PANK3* encodes a protein belonging to the pantothenate kinase family. The pantothenate kinase is a regulatory enzyme that plays a key role in the biosynthesis of coenzyme A (CoA) in bacteria and mammals. It catalyzes the first key step leading to the common biosynthesis of coenzyme A in cells. In detail, PANK3 acts as an inhibitory regulator of intracellular CoA that is produced in the cytoplasm of acetic acid [46]. The acetic acid is one of the two sources of 4-carbon units that are initially synthesized by mammary fatty acids (the other is β-hydroxybutyric acid), and acetyl-CoA provides carbon atoms for the extension of the carbon chain during fatty acid synthesis. Overexpression of miR-200c reduced the expression of the target gene PANK3, thereby releasing the inhibitory and regulating effect of PANK3 on acetyl-CoA, and promoting acetyl-CoA to provide more substrates for fatty acid synthesis, resulting in an increased synthesis of *de novo* fatty acid in ovine MECs. These findings revealed that miR-200c promotes the triglycerides synthesis in ovine MECs via increasing the lipid synthesis related genes expression by targeting *PANK3*. However, it was worth noting that a miRNA can target multiple genes to play its biological function. This means that miR-200c may also regulate the synthesis of triglycerides through targeting other target genes, but this needs to be further explored.

## 4. Materials and Methods

### 4.1. Ethics Statement

Experimentation on the sheep was carried out according to the guidelines for the care and use of experimental animals, as established by the Animal Care Committee of Gansu Agricultural University, Lanzhou, China (Approval number GSAU-ETH-AST-2021-027).

### 4.2. Collection of Ovine Tissue Samples

A total of six healthy, three-year-old, fourth parity Small-tailed Han sheep were selected for investigation at the Jinzihe Sheep Breeding Company in Tianzhu County, People’s Republic of China. All these ewes were raised under the same environmental conditions for natural light and free access to food and water. The parenchyma of mammary gland was firstly collected according to the surgical method [47], including non-lactation, pre-pregnancy at day 45 of pregnancy, late pregnancy at day 140 of pregnancy, pre-lactation at day 15 of lactation, mid-lactation at day 30 of lactation, and late-lactation at day 60 of lactation. Moreover, other tissues were also collected at mid-lactation period after slaughter (*n* = 3), including longissimus dorsi muscle, heart, liver, kidney, spleen, lung and ovary. These samples were utilized to analysis the expression of miR-200c using reverse transcription quantitative PCR (RT-qPCR).

### 4.3. Isolation and Culture of Ovine MECs

Ovine MECs were cultured in DMEM/F12 growth medium (Gibco, Carlsbad, CA, USA), containing 5 μg/mL 17-β-estradiol, 10 μL/mL insulin–transferrin–sodium selenium, 5 μg/mL hydrocortisone and 10 ng/mL epidermal growth factor and 10% fetal bovine serum (FBS) (Gibco, New York, NY, USA), and then incubated at 37 °C in a humidified atmosphere of 5% CO_2_ according to the isolation method with our previously described [48]. After removing fibroblasts according to the previously described method [48], the high-quality ovine MECs were obtained, and 2 μg/mL of prolactin was added at the beginning of the experiment to stimulate the secretion of milk liquid from the ovine MECs.

### 4.4. Transfection of RNA Oligo

For miRNA overexpression, ovine MECs were transiently transfected with either the mimic NC or miR-200c mimic (50 nM, RiboBio, Guangzhou, China) using a INVI DNA & RNA Transfection Reagent™ (Invigentech, Carlsbad, CA, USA). Similarly, for the miRNA knockdown, cells were transfected with either the inhibitor NC or miR-200c inhibitor (100 nM, RiboBio).

### 4.5. Cell Proliferation Assay

The ovine MECs were seeded to 48-well plate with a number of 2 × 10^5^ cells, and miR-200c mimic/inhibitor or mimic/inhibitor NC with 3 repetitions were transfected into MECs when cells were at 70% confluence. For CCK8 assay, the ovine MECs were seeded to 48-well plate containing 20 μL CCK8 solution for 2 h at 37 °C, and followed by measuring absorbance at 450 nm after treatment for 46 h. For EDU imaging assay, the ovine MECs were treated with 50 μmol/L EDU medium for 4 h after transfection for 44 h. Then the cells were fixed with 4% paraformaldehyde, they were stained with Apollo reaction solution. Subsequently, cell nucleus was stained with Hoechst 33,258 (Solarbio, Beijing, China), while the nucleus of proliferation cells was stained with Edu using the BeyoClick™ EdU-555 Kit (Beyotime, Shanghai, China) according to the instructions. Finally, the results were visualized using a biological microscope IX73 (Olympus, Tokyo, Japan), and the data were analyzed using Image J v1.8.0 software (USA).

### 4.6. Target Predictions and Luciferase Activity Assay

The relationship between the binding sites of miR-200c and target genes were identified using miRanda 3.3a and TargetScan 3.1, and then the results of prediction from the two kinds of software were overlapped. According to combination sequence of the seed sequence of the mature miRNA with the sequence of the 3′UTR region of the target gene, pairs of primers (Table S1) were designed to construct wild-type (WT) and mutant (MUT) dual-luciferase reporter vectors using NotI and XhoI restriction sites (Promega, Madison, WI, USA). Subsequently, a 0.5 μg of WT/MUT dual-luciferase vector and miR-200c mimic were respectively co-transfected into HEK293T cell s at 70% confluences, and corresponding control groups were also established. The luciferase reporter assay system (Promega, Madison, WI, USA) was utilized to assess the luciferase activity after 48 h of transfection.

### 4.7. RT-qPCR and Western Blot

The total RNA from the cells at 48 h after transfection and tissues was isolated using a Trizol reagent (Invitrogen, Carlsbad, CA, USA), and the quality of total RNA was assessed by a NanoDrop 2000 (Thermo Scientific, Waltham, MA, USA) and Agilent 2100 (Agilent, Palo Alto, CA, USA) instruments. The high-quality cDNA was synthesized using FastKing gDNA Dispelling RT SuperMix (Tiangen, Bejing, China) for analyzing mRNA expression, whilst the miRNAs was synthesized using the Mir-X Kit (Takara, Tokyo, Japan) for analyzing miRNA expression. All primer was designed using Primer 5.0 according to sequences published on the NCBI databases (Table S1). The GAPDH and U6 gene as reference genes were used to correct the expression of mRNA and miRNAs. The RT-qPCR was implemented on the Applied Biosystems QuantStudio*^®^*6 Flex (Thermo Lifetech, Waltham, MA, USA) system using SYBR Premix Ex Taq II (Takara, Dalian, China) and miRcute miRNA qPCR Detection Kit (Tiangen, Beijing, China). Their relative expression was quantified using a 2^−ΔΔCt^ method [49].

The total proteins were extracted from cells using RIPA, and extracted total proteins were then quantified using a BCA kit (Solarbio, Beijing, China). The total proteins were separated via 12% SDS-PAGE (Solarbio, Beijing, China). Then, the proteins were transferred to a PVDF membrane (Millipore, Burlington, MA, USA) for 1 h. The PVDF membrane was blocked in a 5% skim milk powder-Tris-buffered saline with Tween-20 solution at room temperature for 3 h and then incubated with a primary anti-PANK3 antibody (1:500, Proteintech, Wuhan, China) and an anti-actin antibody (1:1000, Proteintech, Wuhan, China) overnight at 4 °C. Subsequently, the PVDF membrane was washed with Tris-buffered saline with Tween-20 (10 min/wash, 3 times in total) and then incubated with a secondary antibody (goat anti-rabbit IgG, Proteintech) at room temperature. Chemiluminescence substrates (Tanon, Shanghai, China) were used to visualize the protein bands, and the ImageJ program was used for quantification.

### 4.8. Triglycerides Content Detection

When a 50 nmol/L miR-200c mimic and mimic NC or 100 nmol/L miR-200c inhibitor and inhibitor NC were transfected into ovine MECs for 48 h, the content of intracellular triglycerides was measured using a cell triglycerides assay kit (Solarbio, Beijing, China) according to the manufacturer’s recommended protocol. All the experimental data were shown as the mean ± standard deviation. Significant differences were evaluated through *T*-test using SPSS 22.0 software (SPSS Inc., Chicago, IL, USA). The difference between groups was indicated at *p* < 0.01 or *p* < 0.05.

## 5. Conclusions

In ovine MECs, miR-200c specifically binds the *PANK3* and affects milk fat synthesis. Therefore, miR-200c and *PANK3* may be used as a candidate miRNA and target gene respectively, for studying milk fat metabolism in sheep. This study provides a better understanding of the functions of miR-200c in mammary gland growth and development.

## Data Availability

The data presented in this study are available in the article and Supplementary Material.

## Supplementary Materials

The following supporting information can be downloaded at: https://www.mdpi.com/article/10.3390/ijms232415601/s1.
