# Supplementary material for: MicroRNA-200c Affects Milk Fat Synthesis by Targeting PANK3 in Ovine Mammary Epithelial Cells
Source: Int J Mol Sci. 2022 Dec 9;23(24):15601. doi: 10.3390/ijms232415601 (PMC9779841; doi:10.3390/ijms232415601)
Supplement: Supplementary file 1 [file ijms-23-15601-s001.zip › ijms-2006358-supplementary.pdf]

Table S1. Sequence information of primers designed for PCR and RT-qPCR

Name	**Primer**	**Sequence**	**Amlicon size (bp)**	**Gene ID**	**Purpose of primers**
PANK3 (WT)	Forward	CgagctcGGGTTCTGTGCTCTTTCT	836	XM_004016853.5	pmiR-RB-Report™
	Reverse	CCGctcgagTTTGGCTGAAGTATCTGG			
PANK3 (MUT)	Forward	CTGAccgtcataaGCAGAATTATGAAAGGATCATTGTATATT			
	Reverse	CTGCttatgacggTCAGATTCCCACAGTTTCAAAACC			
PANK3	Forward	GATGAACTTGACTGCCTTGT			RT-qPCR
	Reverse	TCTTTGGAATGGACTGCT			
FABP4	Forward	TGTCCTTCAAATTGGGCCAGG	187	NM_001114667.1	
	Reverse	AGCAGTGACACCGTTCATGAC			
LPL	Forward	ACCTGAAGACTCGTTCTC	176	NM_001009394.1	
	Reverse	CACCTCCGTGTAAAGTAG			
ACACA	Forward	GTCCTCTGCCAGTTTCCC	173	NM_001009256.1	
	Reverse	TCCATCACCACAGCCTTC			
GAPDH	Forward	ATCTCGCTCCTGGAAGATG	113	NM_001190390.1	
	Reverse	TCGGAGTGAACGGATTCG			
MiR-200c	Forward	TAATACTGCCGGGTAATGATGG			
	Reverse	/			
U6	Forward	ACGGACAGGATTGACAGATT			
	Reverse	TCGCTCCACCAACTAAGAA			

Note：WT: The primers used for constructing wild-type (WT) pmiR-RB-Report™ vector.

MUT: The primers used for constructing mutated (MUT) pmiR-RB-Report™ vector.
